# De-extinction technology and its application to conservation

**DOI:** 10.1093/jhered/esaf069

**Published:** 2025-09-24

**Authors:** Stephen D Turner, Anna Keyte, Andrew Pask, Beth Shapiro

**Affiliations:** Colossal Biosciences, Dallas, TX, United States; School of Data Science, University of Virginia, Charlottesville, VA, United States; Colossal Biosciences, Dallas, TX, United States; Colossal Biosciences, Dallas, TX, United States; School of Biosciences, The University of Melbourne, Parkville, Victoria, Australia; Museums Victoria, Melbourne, Victoria, Australia; Colossal Biosciences, Dallas, TX, United States; Department of Ecology and Evolutionary Biology, University of California Santa Cruz, Santa Cruz, CA, United States

**Keywords:** conservation, de-extinction, genome engineering, CRISPR, artificial intelligence (AI), biodiversity conservation

## Abstract

De-extinction, once the realm of science fiction, has evolved into a tangible scientific endeavor thanks to breakthroughs in genome sequencing, engineering, advanced assisted reproductive technologies, and stem cell biology. Alongside this work are innovations in reintroduction science and artificial intelligence, which are refining strategies for species translocations, rewilding, and long-term ecosystem monitoring of de-extinct species and populations. While the primary motivation for de-extinction is restoring lost ecological functions to eroded ecosystems, each of these technologies can also be applied to conservation biology for de-endangerment, offering new solutions for biodiversity preservation. This review synthesizes the technological advancements emerging from de-extinction science and explores their broad applications in conservation, demonstrating how de-extinction is both about resurrecting lost species and about expanding the conservation toolkit to sustain and rebuild biodiversity in the face of accelerating environmental change.

## 1. Introduction

The current biodiversity crisis is considered by many to be the sixth major extinction (Kolbert 2024), with loss of functional and phylogenetic diversity almost entirely driven by anthropogenic factors (Matthews et al. 2024). The International Union for Conservation of Nature (IUCN) estimates that more than 46,300 species are threatened with extinction (IUCN 2025), with global meta-analyses showing that immediate action is needed to halt genetic diversity loss (Shaw et al. 2025). De-extinction is the notion that long-dead species can be brought back from extinction through modern genomic techniques and assisted reproductive technologies (ART). The idea of de-extinction broadly captured the public imagination with the publication of *Jurassic Park* 35 years ago (Crichton 1990). Since then, researchers in ancient DNA, genome engineering, embryology, animal husbandry, and ecology have transformed de-extinction from a speculative idea into a tangible scientific pursuit (Shapiro 2017; Novak 2018). The first partially successful de-extinction was the bucardo (*Capra pyrenaica pyrenaica*, also known as the Pyrenean ibex), which went extinct in 2000. A calf cloned from preserved bucardo cells was born in 2003, but survived only briefly (Folch et al. 2009). A selection of previous and ongoing de-extinction projects is provided in Table 1.

**Table 1:** Past and ongoing de-extinction projects. BP = before present; SCNT = somatic cell nuclear transfer.

Species	Extinction date	Original habitat	De-extinction technique	Ecological role	Project leaders	Conservation benefits to extant species
**Pyrenean Ibex a.k.a. Bucardo (*Capra pyrenaica pyrenaica*)**	2000 (original extinction) & 2003 (death of first de-extincted animal)	Pyrenees	SCNT using domestic goat as surrogate	Alpine ecosystem regulator (prevent vegetation overgrowth), trophic interactions (supports apex predators)	Advanced Cell Technology, University of Zaragoza	Protocol development for interspecies cloning; informs genetic rescue of endangered Iberian ibex subspecies
**Dire wolf (*Aenocyon dirus*)**	~13,000 BP	Plains, grasslands, and forests of North America	CRISPR editing of gray wolf cells, SCNT using a domestic dog surrogate	Apex predator regulating prey populations, maintaining ecosystem balance by hunting large herbivores preventing overgrazing	Colossal Biosciences	Required extensive comparative genomics on other canid species, technological development that will benefit other endangered species including the red wolf.
**Woolly mammoth (*Mammuthus primigenius*)**	~4,000 BP	Arctic tundra	CRISPR editing of Asian elephant genomes	Keystone arctic ecosystem engineer: maintain permafrost stability via vegetation trampling, grassland restoration	Colossal Biosciences, Harvard University	Arctic rewilding model; CRISPR delivery systems applicable to elephant conservation
**Dodo (*Raphus cucullatus*)**	1662	Mauritius forests	Primordial germ cell editing of Nicobar pigeon genomes, interspecies germ cell transplantation	Seed dispersal, nutrient cycling. Restoring the Dodo would require control of invasive species, and habitat protection, benefitting ongoing conservation projects in Mauritius	Colossal Biosciences, Mauritian Wildlife Foundation	Avian cloning techniques benefitting other bird species recovery (e.g. pink pigeon); invasive predator management insights
**Thylacine a.k.a. Tasmanian tiger (*Thylacinus cynocephalus*)**	1936	Tasmanian woodlands	Multiplex CRISPR editing of fat-tailed dunnart genomes	Apex predator controlling endemic and invasive mesofauna	Colossal Biosciences, University of Melbourne TIGRR Lab	Marsupial stem cell advances aiding other marsupial conservation (e.g. quolls)
**Quagga (*Equus quagga quagga*)**	1883	South African grasslands	Backbreeding of plains zebras	Grassland management (preventing overgrowth and promoting diversity), keystone herbivore.	The Quagga Project	Selective breeding framework applicable to endangered equids
**Aurochs (*Bos primigenius*)**	1627	European forests and grasslands	Backbreeding of Heck cattle with other European breeds	Megaherbivore keystone grazer and ecosystem engineer. Prevents excessive tree growth, maintaining biodiverse habitats.	The TaurOs Programme (Rewilding Europe and Grazelands Rewilding)	Habitat engineering benefits European bison; promotes rewilding research
**Passenger pigeon (*Ectopistes migratorius*)**	1914	North American forests	CRISPR editing of band-tailed pigeon genomes	Forest regeneration via seed dispersal, nutrient cycling, growth promotion via droppings	Revive & Restore	Avian cloning techniques benefitting other bird species, flock dynamics studies applicable to other endangered avian species
**Heath hen (*Tympanuchus cupido cupido*)**	1932	Coastal Northeast America	CRISPR editing of prairie chicken primordial germ cells	Ecosystem maintenance via foraging, disturbing vegetation, and seed dispersal, trophic interactions (supports predators)	Revive & Restore	Avian cloning techniques, PGC culture techniques, genetic rescue template
**Gastric brooding frog (*Rheobatrachus* spp)**	1983 (southern) 1987 (northern)	Eastern Australia	SCNT	Mid-level aquatic predator regulating insect and invertebrate populations in rainforest stream ecosystems.	Lazarus Project	Amphibian cloning techniques developed for this project are directly applicable to conservation of other critically endangered amphibians.

**Fig. 1:**
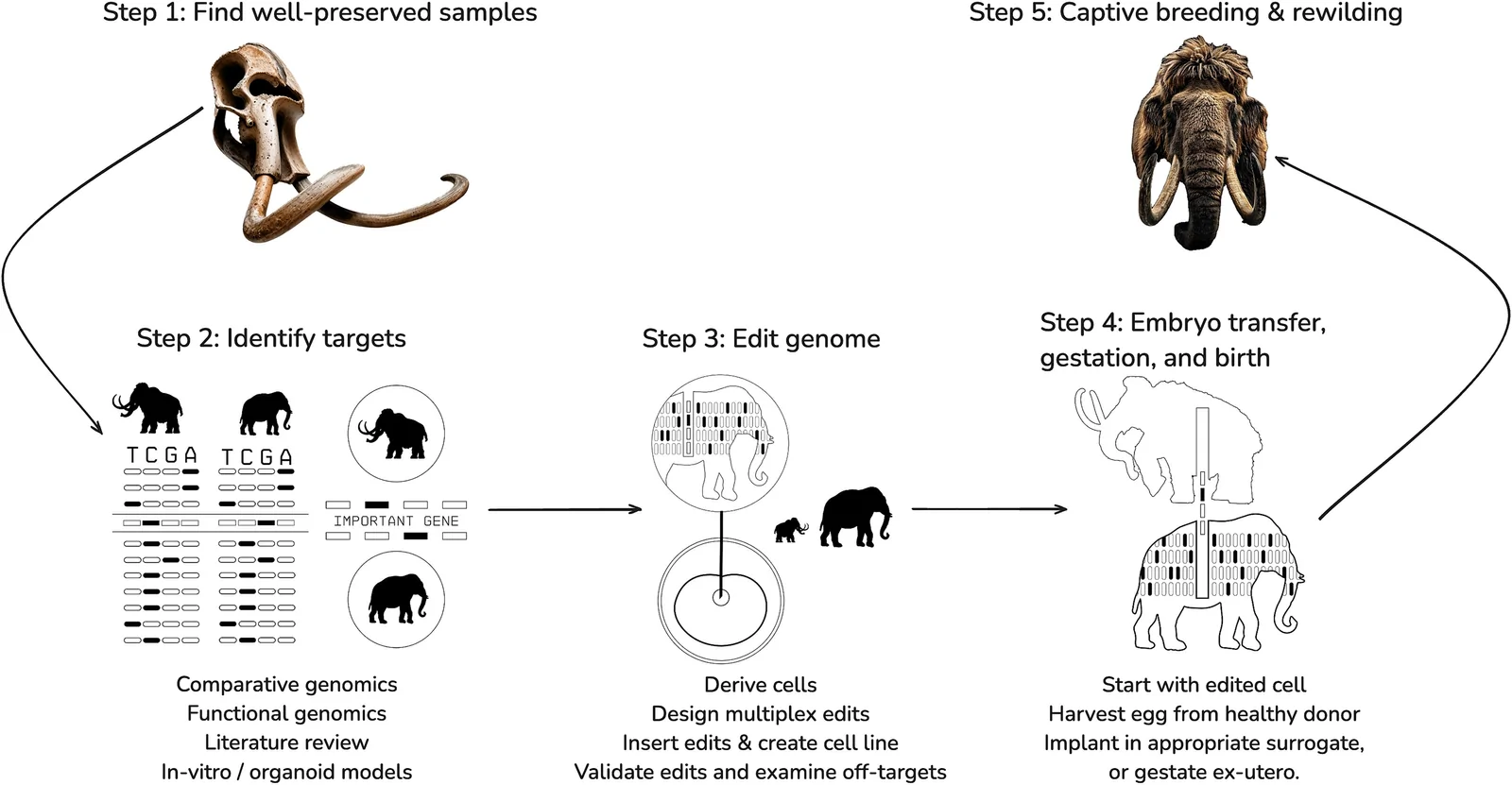
A schematic overview of the de-extinction process. Step 1 involves recovering and sequencing well-preserved ancient samples. Step 2 uses comparative genomics, functional assays, literature reviews, and experiments in model systems to pinpoint key genetic targets and the phenotypic traits they control. Step 3 employs genome editing tools to introduce these edits into living cells. Step 4 entails embryo transfer and gestation in a suitable surrogate (note this illustration is for mammalian species; birds require a different approach of editing and transplanting primordial germ cells). Finally, step 5 focuses on raising and breeding the resulting de-extinct proxy offspring in captivity, followed by their eventual reintroduction into the wild to fill the lost ecological role of the original extinct animal. This final step requires active collaboration with local partners and governments, and extensive monitoring of reintroduction efforts to evaluate and enhance positive ecological impacts.

De-extinction can be understood as a continuum where at one end lies an organism whose genome, phenotype, and ecological role are indistinguishable from the original and, at the other, a functionally equivalent proxy of the extinct species that is capable of restoring lost ecological functions (IUCN 2016; Shapiro 2017; Novak 2018). De-extinction can proceed via different paths. Selective back-breeding, for example, is being used to restore the ancestral traits that defined aurochs, the wild ancestor of domestic cattle (Stokstad 2015). Back-breeding, however, takes many generations and requires living relatives that preserve the targeted extinct traits. Another approach is somatic cell nuclear transfer (SCNT) or ‘cloning’, which creates an identical copy of an extinct organism from a cryopreserved nucleus by inserting it into an enucleated egg of a suitable surrogate. This approach was used for the bucardo and to de-extinct the gastric brooding frog (Novak 2018). Cloning, however, requires an intact frozen cell, and this approach may not be possible for birds and reptiles due to the inability to access the nucleus at the stage required. A third approach, genome engineering, alters the genome of a living organism to add or replace DNA sequences with the goal to engineer extinct phenotypes. Each of these approaches can lead to restoration of traits that defined the extinct species in a living surrogate (Fig. 1).

This review first explores how recent technological breakthroughs have propelled de-extinction research into a new phase. The rapid evolution of sequencing, assembly, and annotation methods provides increasingly precise reconstruction of extinct species’ genomes. Innovations in protein engineering and precision genome editing have equipped researchers to efficiently modify genomes. New developments in ART and ex-utero development (i.e., ‘artificial wombs’), animal husbandry, and ecological modeling of rewilding efforts are priming the path toward birth, release, and management of de-extinct species.

**Fig. 2:**
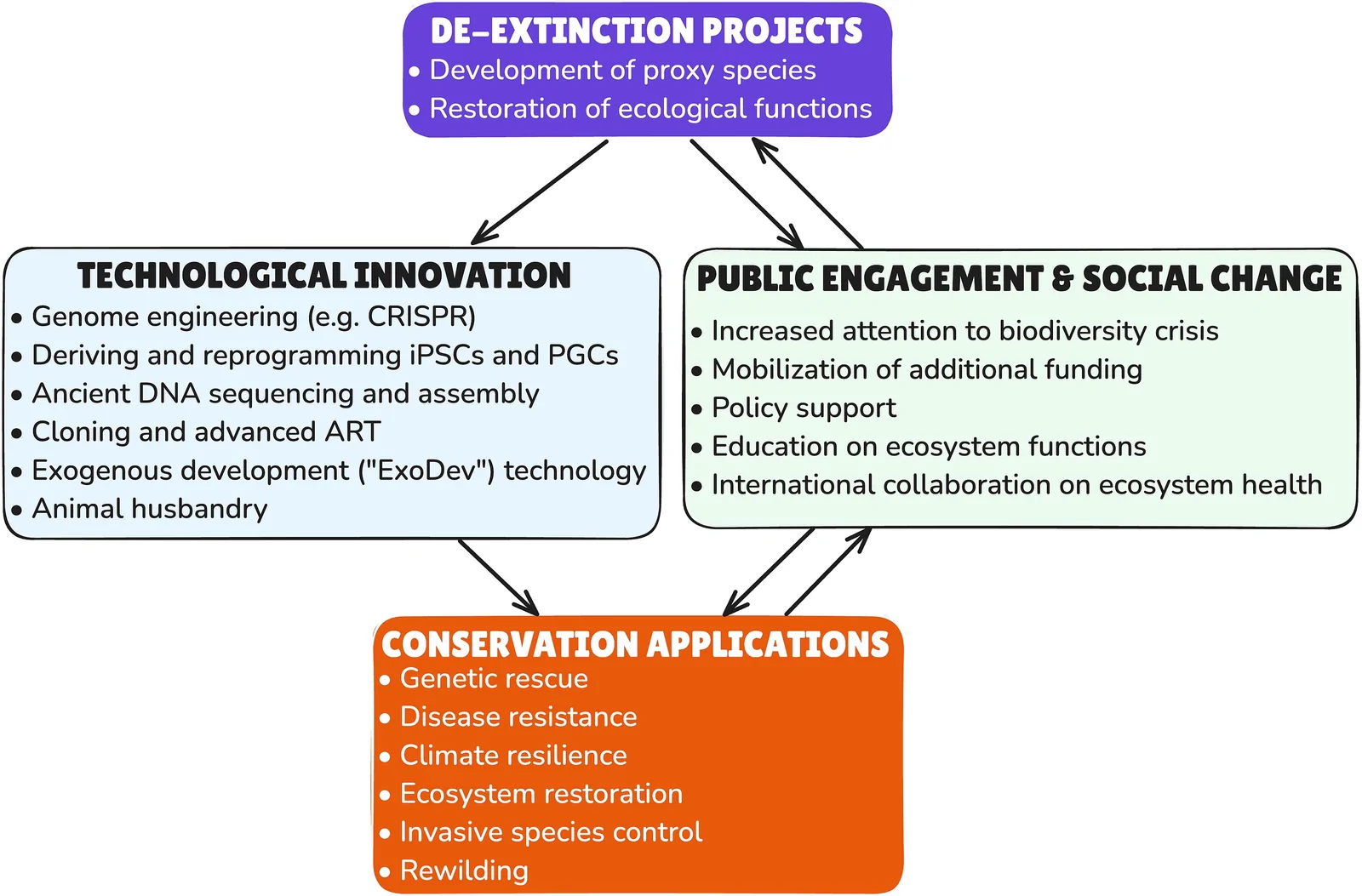
The de-extinction + conservation flywheel. De-extinction efforts reinforce conservation programs by developing new technologies and increasing public engagement and support for conservation programs. De-extinction projects aiming to create proxy species to restore lost ecological functions result in both technological innovation and increased public engagement on the biodiversity crisis. These technological advancements, along with public attention tied to increased funding and policy support both have positive benefits on conservation applications that can take advantage of de-extinction technologies. The newly focused attention and technology brought to bear on difficult conservation programs results in further increased public engagement and funding + policy support, benefitting both de-extinction and conservation applications.

The second half of this review explores benefits that de-extinction research is bringing to conservation, with a focus on vertebrate species. The de-extinction + conservation flywheel illustrated in Fig. 2 describes how de-extinction research leads to new technologies, increases public engagement, and mobilizes funding to benefit conservation efforts. The ethical considerations of de-extinction have been discussed at length elsewhere (Sherkow and Greely 2013; Donlan 2014; Jones 2014; IUCN 2016; Seddon and King 2019; Macfarlane et al. 2022; Odenbaugh 2023; Lean 2024; Molhuizen et al. 2025), and include, for example, the potential diversion of resources from extant species conservation, moral hazard effects, and uncertainty about how proxies will perform in present-day ecosystems. By situating de-extinction technologies within this broader context, this review aligns with IUCN Species Survival Commission guiding principles for de-extinction, which emphasize risk assessment, long-term monitoring, and adaptive management (IUCN 2016).

The accelerating rate of biodiversity loss has created an urgent need for innovative conservation solutions (Shaw et al. 2025). As ecosystems become increasingly fragmented and degraded, the potential to use de-extinction technologies to restore key ecological functions offers a promising avenue for conservation intervention. By exploring both the scientific underpinnings and the practical applications of these emerging technologies, we hope to illuminate the dual promise of de-extinction research: restoring lost ecosystem functions while forging innovative conservation solutions for species currently threatened with extinction today.

## 2. Part 1: Technology

This section explores how technical steps in the de-extinction process, including genome engineering, advanced reproductive technologies, and ecological modeling, are reshaping both de-extinction possibilities and conservation practice. Once genomic loci underpinning the target phenotype are known, the donor genome needs to be engineered to contain these edits. This step requires an advanced genome engineering toolkit that includes, in some cases, the synthesis and delivery into the cell of long DNA fragments. Once the cell’s genome is engineered, the nucleus containing the engineered genome must be transformed first into an embryo and eventually into a living animal, which requires the development of a suite of advanced assisted reproductive technologies including, perhaps, entirely exogenous artificial wombs. Finally, engineered organisms need to be introduced into present-day habitats, which requires tools to monitor the immediate and long-term health of the animals and the ecosystem. These technological advancements are not only making de-extinction feasible, but are delivering benefits that reinforce and expand the modern conservation toolkit.

### 2.1. Genome engineering

Genome engineering has evolved from labor-intensive methods to highly efficient and multiplexable platforms capable of precise edits at scale. Foundational tools like zinc finger nucleases (ZFNs) and TALENs established the principle of programmable DNA cleavage (Urnov et al. 2010; Joung and Sander 2013), and CRISPR-Cas9 democratized genome editing and enabled wider adoption, including in conservation biology (Cong et al. 2013; Mali et al. 2013; Adli 2018). This transition also marked a shift from targeting single loci using low-efficiency homology-directed repair (HDR) to increasingly specific and scalable strategies for editing many loci with high fidelity (Adli 2018; Wang and Doudna 2023).

CRISPR's core architecture has since evolved into powerful new modalities (Table 2). Base editing allows for single-nucleotide conversions without double-strand breaks (Komor et al. 2016; Rees and Liu 2018), while prime editing expands the editable range to small insertions, deletions, and substitutions with high precision and minimal off-target effects (Anzalone et al. 2020; Chen and Liu 2023). Recent improvements in pegRNA design and Cas variants are increasing editing efficiency in non-dividing cells (Scholefield and Harrison 2021; Nelson et al. 2022). For large DNA fragment integration, techniques like PASTE (Programmable Addition via Site-specific Targeting Elements), dual prime, and twin prime editing now permit insertion of gene-sized fragments or replacement of entire loci (Anzalone et al. 2022; Yarnall et al. 2023; Zhao et al. 2025). These developments are especially impactful in de-extinction research, where restoring complex traits may require editing tens of thousands of bases. What was once technically out of reach is now becoming tractable, particularly when coupled with advances in long-fragment DNA synthesis and assembly.

**Table 2:** Genome engineering and long DNA synthesis methods used in de-extinction with potential applications in conservation.

	Modality	Original reference(s)	Summary	Key benefits
**Gene Editing**	**Homology directed repair (HDR)**	(Cong et al. 2013; Mali et al. 2013)	Uses a homologous donor template to accurately insert or modify genetic sequences at a targeted site after a double-strand break	Foundation for knock-in models; predictable outcomes with homologous templates
	**Base editing (BE )**	(Komor et al. 2016)	Modified Cas9 fused to a deaminase enzyme to directly C-G to T-A (cytosine BE) or A-T to G-C (adenine BE) without DSB	Single-base resolution; avoids DSB risks; works in non-dividing cells, simpler than PE for specific point mutations
	**Prime editing (PE)**	(Anzalone et al. 2019)	Modified Cas9 fused to a reverse transcriptase and a prime editing guide RNA (pegRNA) to introduce targeted insertions, deletions, or base substitutions without requiring DSBs or donor templates	Compared to HDR more efficient in non-dividing cells and avoids the need for donor DNA, offers greater flexibility, and avoids DSBs
	**PASTE (Programmable Addition via Site-specific Targeting Elements**	(Yarnall et al. 2023)	Combines CRISPR-Cas9 with a recombinase system to enable efficient, targeted insertion of large DNA sequences without relying on double-strand breaks or homology-directed repair	Compared to HDR and PE, PASTE allows targeted insertion of much larger sequences at high efficiency (30 kb+)
	**TwinPE**	(Anzalone et al. 2022)	Two pegRNAs edit distant loci for insertion, deletion, or replacement of very large scale sequences without DSBs.	Compared to standard PE, allows for more efficient and flexible large sequence modification
	**DualPE**	(Zhao et al. 2025)	Prime editor plus two engineered pegRNAs for scarless deletions, replacements, and inversions of large chromosomal segments without DSBs.	Can introduce deletions exceeding 10 kb, demonstrated deletions up to 2 Mb in hexaploid wheat.
**DNA Synthesis**	**Enzymatic DNA synthesis**	(Hoose et al. 2023; Simmons et al. 2023) (Reviews)	Terminal deoxynucleotidyl transferase (TdT)-based methods extend DNA strands one base at a time enzymatically, enabling synthesis of longer, more accurate DNA fragments in aqueous conditions.	Environmentally friendly; on-demand benchtop synthesis; extends reach beyond traditional phosphoramidite chemistry; successful synthesis of > 1000 bp oligonucleotides.
	**Polymerase cycling assembly (PCA )**	(Stemmer et al. 1995)	Overlapping oligonucleotides are repeatedly denatured, annealed, and extended by polymerase in a thermal cycling reaction to assemble full-length DNA constructs without a cloning step.	Straightforward method for de novo gene synthesis from short oligos; scalable; does not require pre-existing templates; widely used in synthetic biology for gene or pathway construction.
	**Golden Gate assembly**	(Engler et al. 2008)	Type IIS restriction enzymes generate user-defined overhangs allowing seamless, directional assembly of multiple DNA fragments in a one-pot ligation reaction.	High efficiency for multiplexed, combinatorial, and hierarchical assemblies; scarless junctions; ideal for standardized and large-scale gene circuit or operon construction.
	**Gibson assembly**	(Gibson et al. 2009)	Isothermal one-pot reaction that joins multiple overlapping DNA fragments by exonuclease resection, annealing, polymerase fill-in, and ligation.	Simple, scarless assembly of multiple fragments in a single reaction; widely used for modular cloning and synthetic gene construction up to tens of kb.
	**Yeast-based hierarchical assembly**	(Gibson et al. 2009; Mitchell et al. 2017)	Assembling long DNA fragments into kilobase-to-megabase scale constructs inside yeast using homologous recombination, stitching overlapping DNA fragments together.	Enables synthesis of very large constructs (up to entire synthetic chromosomes); powerful for complex assemblies requiring dozens of fragments; exploits yeast's natural recombination machinery.
	**Multiplexed Golden Gate pooled assembly**	(Freschlin et al. 2025)	Fragmenting target sequences into Golden Gate-compatible oligos and assembling hundreds to thousands of full-length genes in parallel using Type IIS restriction enzymes and ligation cycles.	Extremely low per-gene cost; scalable parallel assembly of many full-length genes; democratizes access to large-scale synthesis with only standard lab equipment.

DSB = double-strand break; BE = base editing; PE = prime editing; HDR = homology-directed repair; PCA = Polymerase cycling assembly.

Some genome engineering applications are likely to require the synthesis and delivery of long DNA fragments, perhaps spanning entire genes. To this end, emerging enzymatic and chemical DNA synthesis techniques are allowing accurate construction of sequences spanning entire genes and gene clusters (Table 2). Enzymatic DNA synthesis platforms harness terminal deoxynucleotidyl transferase to build complex DNA strands hundreds to thousands of bases with high fidelity (Hoose et al. 2023), recently achieving > 1000 bp oligonucleotides in a single synthesis reaction (Simmons et al. 2023). Chemical synthesis relies on high-throughput phosphoramidite chemistry on microchips combined with efficient assembly to deliver multi-kilobase gene constructs in massively parallel fashion (Hoose et al. 2023).

For the longest fragments, it may be necessary to perform hierarchical assembly of synthesized DNA fragments, which can be achieved using techniques like Gibson assembly (Gibson et al. 2009) and polymerase cycling assembly (Stemmer et al. 1995). Alternatively, in vivo yeast-based recombination can stitch together dozens of fragments into kilobase to megabase constructs. This approach has enabled milestone projects such as bacterial genome synthesis (Hutchison et al. 2016) and synthesis of designer yeast chromosomes (Mitchell et al. 2017). While traditionally slow and expensive, recent innovations in DNA synthesis and assembly are allowing generation of hundreds to thousands of full-length genes in parallel with per-gene costs under $2 USD (Freschlin et al. 2025).

### 2.2. Advanced assisted reproductive technologies

The integration of stem cell technologies, cryopreservation, and assisted reproductive techniques offers critical tools for both de-extinction and contemporary conservation (Hildebrandt et al. 2021; Marx 2025). These tools make it possible, for example, to biobank tissues from endangered species and transform nuclei with edited genomes into embryos and eventually living animals, as well as provide opportunities to increase genetic diversity and population size without relying on traditional breeding. This section reviews the suite of technologies known as advanced assisted reproductive technologies (aART), which includes induced pluripotent stem cell (iPSC) generation, SCNT, and in vitro gametogenesis (IVG), which extend beyond classical techniques like artificial insemination and in vitro fertilization (IVF).

#### 2.2.1. Stem cell applications and reproductive strategies

iPSCs provide versatile platforms for genetic rescue. These somatic cells, reprogrammed to an embryonic-like state by the addition of a small number of transcription factors (Yamanaka 2008), can be generated from endangered species’ tissue and expanded almost indefinitely, circumventing difficulties in obtaining embryos or gametes from rare animals (Hutchinson et al. 2024).

IVG is a particularly exciting application of iPSCs. Obtaining oocytes from endangered species is often a critical bottleneck in ART programs, as ovulation timing tends to be poorly understood and natural oocyte retrieval can be unfeasible. Instead of oocyte retrieval, IVG directs iPSCs to become functional gametes—spermatozoa or oocytes—entirely in a laboratory setting (Saitou and Hayashi 2021), enabling intracytoplasmic sperm injection and embryo creation for transfer into surrogates. Unlike cloning, IVG enables sexual reproduction with meiotic crossover, generating new allelic combinations that increase genetic diversity (Cowl et al. 2024). IVG has been demonstrated in model organisms (Murakami et al. 2023), and is being explored as a way to produce gametes for functionally extinct species like the critically endangered northern white rhinoceros (*Ceratotherium simum cottoni*), where banked sperm exists but viable oocytes are lacking (Hildebrandt et al. 2021; Hayashi et al. 2022; Hutchinson et al. 2024). Recent work to this end has established primordial germ cell-like cells (precursors to gametes) from northern white rhino iPSCs (Hayashi et al. 2022).

iPSCs can also be used to derive spermatogonial stem cells (SSCs). SSCs can be transplanted into the testes of a recipient male of a related species, where they can naturally produce sperm. This approach could enable southern white rhinoceros sires to produce northern white rhinoceros sperm, for example, facilitating natural mating and obviating the need for complex ART procedures.

SCNT is an important aART for conservation. By transferring donor nuclei into enucleated eggs, SCNT can resurrect genomes from deceased individuals, provided viable cells are preserved (Wilmut et al. 2002). While SCNT success rates have improved since the birth of Dolly, the first cloned sheep, challenges remain, as illustrated by the short-lived bucardo clone (Folch et al. 2009) that died due to lung defects potentially caused by interspecies gestational incompatibilities.

Due to fundamental differences in their reproductive biology, birds require a different approach to genetic engineering and cloning. SCNT relies on having access to mature and non-differentiated egg cells. In birds, these cells are only present high in the reproductive tract of hens, which makes them difficult to access without euthanizing the bird. Even if these cells can be accessed, finding and isolating the tiny nucleus of a bird egg cell is extremely challenging—a chicken egg cell is ~10 000 times the diameter of the nucleus. Because of these constraints, aART for avian species relies on germline transmission of cultured primordial germ cells, or PGCs (van de Lavoir et al. 2012; Novak 2018). PGCs are the precursors to sperm and egg cells. After extracting PGCs, they can be propagated in vitro, making their genomes accessible for editing. These PGCs can then be injected into a developing host embryo whose own PGCs have been ablated (Ballantyne et al. 2021). As this first generation (F1) matures, the edited PGCs will form sperm or egg cells. When two of these F1 host animals mate, the F2 zygote derived from edited PGCs will contain all the edits of the genetically engineered PGCs.

This approach can be used to generate both edited and unedited birds and has broad application for genetic rescue and de-extinction. Recently, researchers showed that injecting PGCs from a chicken embryo into a guinea fowl host led to the production of donor-derived sperm, and insemination with this sperm produced normal chicken embryos (van de Lavoir et al. 2012). This suggests that PGC transfer will be useful for applications where a conspecific host species is either extinct or rare. Common or domesticated birds such as chickens, for example, might be used as hosts to boost populations of endangered avian species.

#### 2.2.2. Biobanking and cryopreservation

Biobanking, the cryopreservation of genetic materials (Ryder and Onuma 2018), provides the essential raw materials upon which cloning and stem cell technologies depend, and its expansion goes hand-in-hand with progress in both de-extinction science and conservation biology (Ryder and Onuma 2018).

The San Diego Frozen Zoo was established in 1975 as the first biobank, and was founded to collect DNA, blood, tissue samples, and germplasm from diverse species in anticipation that future technologies might open new possibilities for biodiversity conservation (Benirschke 1984). Today the Frozen Zoo contains over 10 000 living cell cultures, oocytes, sperm, and embryos representing nearly 1000 taxa, with samples duplicated at multiple locations as backups. Since the 1970s, biobanking has expanded into an international network of efforts, with repositories established on every continent. Initiatives such as the Frozen Ark Consortium (frozenark.org), CryoArks in the UK (cryoarks.org), the CryoZoo in Spain (cryozoo.org) and the IUCN Animal Biobanking for Conservation Specialist Group are actively working to catalog and preserve genetic material from thousands of species.

Cryopreserved sperm and oocytes can directly facilitate traditional breeding via IVF, but equally important are cryopreserved somatic cells (e.g. skin fibroblasts) that can be reprogrammed into iPSCs or used for cloning. Biobanks thus safeguard species that are on the brink of extinction and preserve lineages and alleles that might otherwise be lost from the gene pool. Combined with ART, biobanking forms an insurance policy for biodiversity: even if a species dwindles to a few individuals or goes extinct, its frozen cells may enable future revival or augmentation of genetic diversity.

Biobanking and cryopreservation face several challenges. Some cell types, including oocytes, are difficult to freeze and recover with high viability, and collecting materials from wild or endangered animals may not be feasible (Hildebrandt et al. 2021). Biobanked somatic tissues can be transformed into iPSCs, however, offering a valuable alternative. In practice, an endangered species’ genetic repository might consist of frozen tissue biopsies that are later expanded as iPSC lines and used to generate gametes and embryos. While these approaches remain experimental, they offer a path to rescue lost genes or resurrect species with greater genetic diversity by creating multiple distinct individuals from a single source culture) (Hildebrandt et al. 2021; Hutchinson et al. 2024).

Another challenge is the accumulation of chromosomal abnormalities during cell culture, such as aneuploidies observed in African wildcat (*Felis lybica*) fibroblast cell cultures (Gómez et al. 2006). This risk emphasizes the importance of ensuring genome integrity during cell culture passaging, particularly when gene editing is involved, using genomic approaches like whole genome sequencing to detect mutations, aneuploidies, and contamination (Smela et al. 2024).

#### 2.2.3. Interspecies gestation

If a species is extinct or dwindles to a few individuals, interspecies gestation, or implanting embryos into surrogate mothers of related species (Anderson 1988), may be necessary. However, while closely related species can serve as viable surrogates, success rates may be low. Potential causes of incompatibility include immunological rejection, placental dysfunction, or mismatches in gestation length and fetal growth rates (Anderson 1988; Hammer et al. 2001; Elliot and Crespi 2006). Despite these challenges, interspecies SCNT has succeeded in several wildlife species, including the endangered gaur (*Bos gaurus*), mouflon (*Ovis orientalis musimon*), African wildcat (*Felis lybica*), gray wolf (*Canis lupus*), sand cat (*Felis margarita*), and Russian sturgeon (*Acipenser gueldenstaedtii*), and others (Hildebrandt et al. 2021). Recently, a southern white rhinoceros female, Curra, acted as an interspecies maternal surrogate for a northern white rhinoceros embryo produced via IVF and ET (Hildebrandt et al. 2023). Although Curra succumbed to a bacterial infection prior to delivering the calf, this success highlights the power of interspecies gestation to potentially reconstitute self-sustaining populations and increase genetic diversity.

#### 2.2.4. Exogenous development with artificial wombs

Exogenous development (ExoDev), or artificial womb technology, has been proposed as a gestational tool when suitable surrogates are unavailable. ExoDev technology has a long history (Ireland 2024). The first U.S. artificial uterus patent was issued over 70 years ago (Greenberg 1955) and early trials in 1958 kept pre-viable human fetuses alive for up to 12 hours by oxygenating blood through the umbilical cord while submerged in synthetic amniotic fluid (Westin et al. 1958). Later studies successfully transferred lambs from maternal uteri to "artificial placentas" for 40 minutes (Callaghan et al. 1963) and eventually several days (Zapol et al. 1969). Recently, researchers supported a fetal lamb for 4 weeks in a ‘biobag’ filled with a nutrient-rich fluid and gas exchange capabilities (Partridge et al. 2017). Another breakthrough supported mouse embryos from days 5 to 11, which is approximately half their gestation cycle (Aguilera-Castrejon *et al.*, 2021).

While these advances are promising, full term gestation has not yet been achieved. Primary areas for research include replicating the intricate maternal-fetal interface, and establishing functional placentas capable of nutrient exchange, gas diffusion, and immune signaling processes (Ander et al. 2019). Scalability requires supporting growth from microscopic fertilized eggs to fully developed neonates, a process requiring precise physiological and biomechanical adaptations (Ireland 2024). Species-specific developmental cues, including hormonal regulation and immune tolerance, must be mimicked to prevent developmental abnormalities. Additionally, ExoDev systems must provide maternal signals guiding organogenesis, neurological development, and epigenetic programming, all of which are critical for survival and ecological fitness (Hsiao and Patterson 2012; Dimasuay et al. 2016). Given these complexities, ExoDev remains a challenge requiring substantial breakthroughs in developmental biology, biomaterials, and bioengineering before reliably supporting species revival or population restoration efforts. Nonetheless, it is an exciting avenue for research with tremendous potential for both conservation and de-extinction, should these technologies become available.

### 2.3. Reintroduction, rewilding, and monitoring

Successful live birth is the first stage in de-extinction. The second stage includes reintroduction of de-extinct species into wild habitats, the broader conservation strategy of rewilding, and monitoring both the reintroduced species and the rewilded habitat to detect both intended and potentially unintended consequences of the conservation action. While this second stage has not yet been attempted with a de-extinct species, recent advances in traditional conservation programs can guide this next phase.

#### 2.3.1. De-extinction as translocation

The ecological benefits of a de-extinct species are realized after release into the wild (Seddon et al. 2014a). For this reason, it is helpful to consider de-extinction as a form of translocation, which allow de-extinction science to leverage established conservation translocation frameworks. The IUCN's guidelines for reintroductions and conservation translocations (IUCN 2013) emphasize feasibility, risk assessment, and the elimination of original extinction causes. These principles translate directly to de-extinction candidate selection: whether suitable habitat exists under current climate conditions, if extinction factors (habitat loss, invasive predators, disease) have been eliminated, and whether reintroduction would cause ecological disruption (Iacona et al. 2017; Steeves et al. 2017).

The goal of functional de-extinction is to engineer organisms able to perform their ecological roles in current environments (IUCN 2016; Shapiro 2017; Novak 2018). De-extinct species could resume seed dispersal for vanished fruit-eating birds or restore predation pressure lost with extinct carnivores. Of course, today’s ecosystems are not static remnants of the past. Climate change and introduced species have created novel environments (Seddon et al. 2014b), and reintroducing locally extinct (via translocation) or entirely extinct (via de-extinction) species must account for these changes. For example, reintroducing a keystone herbivore requires ensuring its food sources exist and that reintroduction will not harm other endemic species. While deploying translocation or de-extinction requires risk–benefit analysis grounded in accepted science (IUCN 2013; Seddon and Redford 2025), when implemented prudently, de-extinction, like translocation, could enrich biodiversity, restore lost functions, and enhance ecosystem resilience.

#### 2.3.2. Rewilding

While reintroduction is the process of releasing a species into a habitat where it previously existed, rewilding aims to restore entire ecosystems by reinstating natural processes. The goal of the aurochs backbreeding project, for example, is to restore the grazing dynamics that shaped European landscapes for millennia (Jepson 2025). These efforts embrace dynamic, self-sustaining ecosystems that are adaptable to changing climate conditions. In Mauritius, the translocation of giant tortoises (*Aldabrachelys gigantea*) that are functionally analogous to the extinct Mauritian giant tortoise (*Cylindraspis inepta)* is helping control invasive plants (Griffiths et al. 2013). In Europe, researchers are mapping rewilding opportunities across abandoned farmland adjacent to protected zones so that management policies can integrate rewilding to enhance biodiversity conservation (Ceaușu et al. 2015).

Large-scale rewilding projects, like Pleistocene Park in Siberia, intend climate benefits alongside biodiversity restoration. The goal of Pleistocene Park is to restore mammoth steppe ecosystems by replacing unproductive northern ecosystems with highly productive pastures featuring high animal density and accelerated carbon cycling (Zimov 2005; Lorimer et al. 2015). Early results show that herbivore rewilding reduces soil moisture and permafrost temperature, sequesters carbon in soils, and increases regional albedo through surface grassland regeneration (Zimov et al. 2012). Developing scientific frameworks and assessment rubrics will improve the impact of rewilding efforts such as Pleistocene Park on trophic complexity restoration and ecosystem resilience (Perino et al. 2019).

The technological and conceptual advances emerging from reintroduction and rewilding efforts are reshaping conservation strategies. This research is helping refine our understanding of ecological roles, improve genetic and reproductive technologies for endangered species, and develop scalable strategies for restoring ecosystem functions. These insights directly benefit traditional conservation by informing species recovery programs, habitat restoration, and climate adaptation strategies, ensuring that rewilding efforts contribute to broader biodiversity and ecosystem resilience goals.

#### 2.3.3. Artificial intelligence–enhanced modeling and monitoring

Conservation scientists are increasingly leveraging artificial intelligence (AI) and advanced modeling to predict how reintroduced or de-extinct animals might behave in modern ecosystems and to monitor their performance post-release. Machine learning and simulation models can analyze vast amounts of data on animal ecology to forecast movement patterns, habitat use, and species interactions. All of this is vital information for planning successful reintroductions.

#### 2.3.4. Predictive modeling for reintroduction planning

AI techniques are enhancing traditional ecological models by identifying complex patterns beyond the scope of simpler models. Neural networks and machine learning methods can analyze animal movement data (e.g. GPS tracks) to classify behavioral states and predict likely trajectories (Browning et al. 2018; Wijeyakulasuriya et al. 2020). For de-extinction proxies, these models can be trained on data from extant relatives or ecological analogs to predict movement through landscapes and responses to environmental cues. Before releasing de-extinct aurochs into a reserve, managers could use movement models informed by data from cattle to predict whether animals will remain within boundaries or roam, guiding fence design or corridor creation. Similarly, mammoth rewilding could be informed by AI research in elephant monitoring, tracking, behavior analysis, and bioacoustic event detection (Bermant et al. 2022; Brickson et al. 2023; McNutt et al. 2024).

AI-driven modeling also anticipates ecological interactions of reintroduced species, including diet preferences, competition, and effects on vegetation or prey populations. For example, an model incorporating forest composition, food availability, and pigeon bioenergetics was used to explore passenger pigeon (*Ectopistes migratorius*) reintroduction scenarios to identify conditions leading to survival versus starvation (Elzinga et al. 2020). Such experiments allow refinement of reintroduction strategies without risking animal welfare or ecosystem consequences. Similar models could predict how a resurrected mammoth might interact with environments or how de-extinct predators would impact prey populations.

Habitat suitability and niche modeling are another key application (Lahoz-Monfort and Magrath 2021). For extinct species, current and historical occurrence data from fossils or museum records can be combined with environmental layers to predict suitable habitats. AI could integrate data on human presence and infrastructure to predict conflict zones, allowing proactive mitigation measures like community outreach or strategic fencing.

#### 2.3.5. Real-time monitoring and adaptive management

AI-based technologies can augment traditional monitoring by enabling real-time observation of rewilding outcomes and more responsive management interventions. Traditional monitoring (tagging animals, camera surveys, field observation) can be labor-intensive and slow. AI-based technologies could allow observing rewilding outcomes in real time, enabling more responsive and informed management interventions. Innovations include real-time tracking with GPS/satellite collars (Mata et al. 2021) and integration of that data through smart platforms. Modern collars can continuously transmit an animal's location and physiological data. AI algorithms could analyze these incoming streams and detect patterns or anomalies (Kumar and Jakhar 2022). Platforms like EarthRanger, for example, aggregate live data from GPS collars, cameras, and satellite feeds into integrated dashboards, allowing managers to view reintroduced animals’ positions relative to threats and receive automatic alerts for immediate intervention (Wall et al. 2024). Such proactive monitoring could increase survival of reintroduced wildlife and reduce human-wildlife clashes.

Cameras and drones equipped with AI are transforming large-scale monitoring. Deep learning image recognition can automatically process millions of camera trap images to detect animals, identify species, and classify behaviors, saving thousands of hours of human effort (Vélez et al. 2023). In a recent study, deep neural networks applied to a dataset of 3.2 million camera trap images from the Snapshot Serengeti dataset, achieved > 93% accuracy in identifying 48 species (Norouzzadeh et al. 2018). AI-driven drones with thermal cameras can locate and identify animals even in dense terrain. An AI-driven drone system used to survey endangered lemurs collected forest data in three 20-minute flights, reported as equivalent to several months of manual survey work (Durrell Wildlife Conservation Trust 2022). Automated acoustic recording and AI classification allows continuous detection of birds in remote environments, providing a powerful complement to visual methods of wildlife monitoring (G. Freeman, 2024). Habitat changes can also be monitored through AI analysis of satellite imagery (Bekmurzaeva et al. 2024), for example to quantify if introduced large herbivores are leading to expansion of grass cover.

AI’s potential for adaptive management lies in synthesizing continuous monitoring data into actionable insights (Ditria et al. 2022). As GPS, camera, and sensor data accumulate, AI models could identify critical gathering points, disease contact risks, or breeding habitat preferences, informing management decisions. By handling multi-modal data streams, AI provides a holistic view of ecosystem recovery, measuring both survival and ecosystem health, such as tracking wolf reintroduction effects on deer densities, vegetation regrowth, and even riverbank conditions, as observed in Yellowstone (Ripple et al. 2025). By automating data collection and analysis, AI enables more adaptive, evidence-based approaches to rewilding, allowing conservation teams to respond in near real-time to reintroduced population needs and quantitatively assess whether de-extinct species deliver expected ecological benefits.

## 3. Part 2: Conservation benefits from de-extinction research

The technologies being developed as part of de-extinction research have immediate utility in conservation biology, offering new pathways for genetic rescue, disease resistance, and ecosystem restoration (IUCN 2016, 2019b, 2019a; Lean 2024; Macfarlane et al. 2022; Novak 2018). These advances create opportunities for "de-endangerment," which is broadly using biotechnology to enhance genetic diversity, bolster pathogen resistance, and improve the resilience of threatened species across both animal and plant kingdoms (Yin et al. 2024; Marx 2025).

De-extinction projects provide opportunities to study the long-term effects of multiplex gene editing, epigenetic stability, and physiological integration in controlled environments. Longitudinal data on health, development, immune function, and behavior from early generations of genome-edited species in managed care can establish baselines that inform future conservation applications while ensuring animal welfare (IUCN 2016). This rigorous monitoring de-risks applications in threatened species by providing carefully documented case studies of how edited genomes function in vivo over time.

The high public profile of de-extinction projects catalyzes broader conservation efforts, attracting new funding and inspiring collaborative efforts among scientists, policymakers, and communities (Donlan 2014; Jones 2014; Bloom 2024; Nature Biotechnology 2025). This creates a conservation flywheel (Fig. 2) where technological advancements and heightened public engagement mutually reinforce biodiversity preservation efforts. The following sections examine how de-extinction technologies can supplement traditional conservation programs, highlighting candidate species that may benefit from these innovations (Table 3).

**Table 3:** Candidate conservation projects benefitting from the potential application of technologies developed for de-extinction.

Species	IUCN Threat Status	Technology	Conservation benefits
**Black-footed ferret (*Mustela nigripes*)**	Endangered	Somatic cell nuclear transfer (SCNT)	Genetic diversity: Restores 3x genetic diversity seen in current populations from cryopreserved tissue; Combats inbreeding depression.
**Chytrid-susceptible amphibians (**e.g. ***Pseudophryne* spp.)**	Critically endangered (**e.g.** the Corroboree Frog)	Transgenic modification Multiplex genome editing with CRISPR-Cas9	Disease resistance; Engineered antibody and antifungal peptide expression; Targets immune response pathways.
**American chestnut tree (*Castanea dentata*)**	Critically endangered	Transgenic modification	Disease resistance; Introduces blight-resistant oxalate oxidase (OxO) transgene from wheat.
**Reef-building corals (**e.g. ***Acropora* spp.)**	Varies (**e.g.** *Acropora* spp. endangered or critically endangered)	CRISPR-Cas9 gene editing, assisted/directed breeding	Environmental stress resilience; identify natural gene variants in existing coral and their microbiomes that confer tolerance to increasing water temperatures.
**Northern quoll (*Dasyurus hallucatus*)**	Endangered	CRISPR-Cas9 gene editing of ATP1A1	Toxin resistance; Introduces heritable bufotoxin resistance; Enables quolls to act as natural biocontrol agents for invasive cane toads.
**Northern white rhinoceros (*Ceratotherium simum cottoni*)**	Critically endangered	Induced pluripotent stem cells (iPSCs) and IVF	Resurrects genetic diversity from cryopreserved cell lines; Prevents progression from functional extinction to total extinction.
**Pink pigeon (*Nesoenas mayeri*)**	Vulnerable; Critically Depleted*	Multiplex genome editing with CRISPR-Cas9	Increase genetic diversity to reduce genetic load; introduces genetic variation from sequenced museum samples, reducing genetic load and genomic erosion.
**Red wolf (*Canis rufus*)**	Critically endangered	Somatic cell nuclear transfer (SCNT) from blood-derived endothelial progenitor cells	Genetic rescue: Adds 25% new founder diversity from three novel cell lines; Provides a non-invasive cloning platform for biobanking and population supplementation; Enhances capacity for rewilding and long-term species persistence.

### 3.1. Genetic rescue and increasing genetic diversity

Genetic diversity is the cornerstone of population resilience and adaptive potential, yet ongoing rapid loss of this diversity (Shaw et al. 2025) compromises long-term evolutionary viability (Kardos et al. 2021; Matthews et al. 2024). When species experience severe bottlenecks, genetic erosion and accumulation of deleterious mutations—known as genetic load—reduces both the immediate fitness of individuals and the population’s adaptive potential (Grossen and Ramakrishnan 2024). In many threatened species, such losses are so pronounced that, even after demographic recovery, the risk of extinction persists due to diminished genetic variability. Such populations can become trapped in extinction vortices in which reduced fitness and increased inbreeding further decreases effective population size (Bosse and van Loon 2022).

Genetic rescue seeks to bolster adaptive potential by reintroducing lost alleles and reducing inbreeding effects (Whiteley et al. 2015). The pink pigeon of Mauritius (*Nesoenas mayeri*) exemplifies both the challenges and potential of this approach The pink pigeon has faced significant population declines due to habitat destruction and invasive species (Jones and Swinnerton 1997). Despite recovering from a population of fewer than 10 individuals to several hundred, the species suffers from extensive runs of homozygosity and high genetic load (Jackson et al. 2022). Pink pigeons are also susceptible to trichomonosis, further threatening its long-term survival (Bunbury et al. 2008). Genome engineering offers targeted reintroduction of lost genetic variants from museum specimens and cryobanked tissues, potentially correcting harmful mutations and restoring alleles that confer fitness benefits (van Oosterhout *et al.*, 2025).

The functionally extinct northern white rhinoceros (*C. simum cottoni*) illustrates genetic rescue using only cryopreserved material. With only two females remaining, biobanked semen and tissue samples retain genetic variation absent from the living population. Advanced ART, including iPSC derivation and in vitro embryo production, are being developed to harness this preserved genetic diversity for creating new individuals (Hildebrandt et al. 2021; Wilder et al. 2024). The black-footed ferret (*Mustela nigripes*) presents a compelling genetic rescue success story. Having started from only seven reproductively viable individuals, the captive breeding colony established by the U.S. Fish and Wildlife Service faces the potential of severe inbreeding (Tibbetts 2022; Novak et al. 2024). In 2020, a black-footed ferret called ‘Elizabeth Ann’ was born following SCNT using black-footed ferret cells biobanked in 1988, representing a lineage that was not present in the captive population (Fritts 2022; Novak et al. 2024). While Elizabeth Ann was unable to reproduce, another individual, Antonia, was cloned from the same biobanked sample and has so far produced two healthy kits, marking the first time a cloned endangered species produced offspring (U.S. Fish and Wildlife Service 2024). This achievement demonstrates how archived genetic material can reintroduce critical variation and reduce genetic load in bottlenecked populations (Wisely et al. 2015).

Research on dire wolf de-extinction has generated parallel benefits for the critically endangered red wolf (*Canis rufus*). Today only around 20 red wolves survive in the wild and all descended from 14 individuals captured to establish a breeding population in the 1970s (vonHoldt et al. 2022a; vonHoldt et al. 2022b). The dire wolf de-extinction project led to development of SCNT protocols refined for canids and novel pluripotent cell line technologies enabling production of two litters of ‘ghost wolf’ pups—clones of animals that remain part of a wild population of red wolves with some admixed coyote ancestry from coastal Louisiana where the founders of the captive red wolf population were captured. As red wolves are gray wolf—coyote hybrids, integrating ghost wolf ancestry into the red wolf breeding program could increase the diversity of preserved red wolf genomic ancestry and alleviate challenges of fitness decline in small populations due to limited genetic diversity.

### 3.2. Disease resistance and climate resilience

Emerging infectious diseases and climate change are rapidly altering ecosystems, prompting conservationists to explore innovative genetic interventions to bolster species resilience. These challenges often interact, creating compound threats that traditional conservation approaches struggle to address effectively.

#### 3.2.1. Pathogen resistance through genome engineering

Chytridiomycosis, caused by the chytrid fungus, devastates amphibian populations globally (Kilpatrick et al. 2010). Researchers at the University of Melbourne are engineering chytrid resistance into Australia's critically endangered Corroboree Frog (*Pseudophryne* spp.), by engineering transgenes expressing antibodies and antifungal peptides in amphibian skin (University of Melbourne 2024). This approach targets the infection mechanism directly, offering hope for species facing imminent extinction from this pandemic pathogen.

Similarly, invasive species can introduce novel toxins that native predators often cannot tolerate. Australian cane toads have devastated endangered northern quoll (*Dasyurus hallucatus*) populations since 1935, as these marsupials lack physiological defenses against bufotoxin. Recent breakthrough work introduced bufotoxin resistance into marsupial cell lines using genome editing, achieving > 45-fold increased resistance (Ibri et al. 2024). This proof-of-concept demonstrates that genetic engineering can create resilience to toxic invasive species while potentially providing a dual benefit, in that resistant quolls could help control cane toad populations through increased predation.

#### 3.2.2. Climate adaptation and forest restoration

Climate change intensifies the need for species that can adapt to rapidly shifting environmental conditions. Targeted genome editing offers promising avenues for developing climate-resilient traits, as demonstrated in forest tree studies showing increased heat and drought tolerance (Cao et al. 2022).

The American chestnut (*Castanea dentata*) is a striking example of successful restoration through pathogen resistance engineering. Once a keystone tree species in eastern North American forests, American chestnuts were functionally eliminated by the invasive fungus, *Cryphonectria parasitica*, which causes chestnut blight. Researchers developed blight-tolerant American chestnut trees by introducing an oxalate oxidase transgene from wheat that breaks down the pathogen's toxic compounds (Powell et al. 2019). This approach enables the tree and fungus to coexist and avoids creating selective pressure for pathogen resistance, providing an evolutionarily stable solution (Powell et al. 2019). This success provides a model for restoring other disease-affected species.

#### 3.2.3. Marine ecosystem resilience

Ocean warming and acidification threaten coral reefs, which provide billions in economic value through fisheries, tourism, and coastal protection while supporting immense biodiversity (Birkeland 2015). Rising sea temperatures trigger mass bleaching events and structural degradation, threatening both the ecological functions and the economic benefits these ecosystems provide (Henley et al. 2024). Ocean warming can lead to mass bleaching events when symbiotic algae are expelled from coral tissues (Hughes et al. 2018). Genome engineering approaches could introduce or enhance heat tolerance genes identified in resistant coral species, potentially increasing susceptible populations' resilience to rising temperatures (van Oppen and Oakeshott 2020; Selmoni et al. 2024). Complementary strategies targeting coral microbiomes offer additional pathways for enhancing thermal tolerance and ecosystem stability (Epstein et al. 2019).

While these individual-level interventions address specific threats to target species, de-extinction technologies also enable broader ecosystem-scale restoration through the reintroduction of keystone species and management of ecological interactions.

### 3.3. Ecosystem restoration and resilience

De-extinction technologies offer transformative potential for ecosystem restoration by reestablishing keystone species and managing ecological interactions that promote habitat recovery and biodiversity enhancement. These approaches extend beyond species revival to address fundamental ecosystem processes and community dynamics.

#### 3.3.1. Trophic cascades and keystone species restoration

The Yellowstone wolf reintroduction demonstrates how restoring apex predators can trigger powerful trophic cascades that reinvigorate ecosystem processes (Ripple et al. 2025). Gray wolf (*C. lupus*) reintroduction to Yellowstone National Park reduced elk herbivory pressure, leading to increased riparian willow biomass and beaver habitat restoration, ultimately reversing erosion and water table disruption (Hobbs et al. 2024; Ripple et al. 2025). This natural experiment demonstrates how single-species interventions can generate landscape-scale ecological recovery.

Such restoration efforts align with natural climate solutions that emphasize ecosystem protection for carbon capture and storage. Some estimates suggest that species such as elephants, bison, wolves, and whales contribute to ecosystem carbon storage on the order of gigatons annually (Schmitz et al. 2023).

#### 3.3.2. Genetic tools for invasive species management

Invasive species remain major obstacles to ecosystem restoration and were implicated in extinctions like the dodo in Mauritius, where introduced rats disrupted native communities (Fair et al. 2023). Reintroducing species into ecosystems dominated by non-endemic invasive species is unlikely to succeed without addressing these fundamental alterations to ecosystem structure.

Genetic technologies offer distinct approaches to invasive species management and biological control (Teem et al. 2020). RNA interference (RNAi), for example, has emerged as a promising biocontrol method for invasive species (Willow *et al.*, 2022). RNAi works by introducing double-stranded RNA molecules that match target genes, leading to their degradation and effectively silencing gene expression. In invasive species control, this can disrupt essential physiological pathways (such as metabolism, reproduction, or digestion) ultimately reducing survival or fertility. However, RNAi typically acts in a limited way: locally and transiently. Another potential approach, gene drives, bias inheritance patterns to spread genes and associated traits rapidly through populations. These approaches show promise for suppressing and even eradicating invasive rodents on islands, where native species are especially vulnerable (Burgess et al. 2021). CRISPR-based gene drives have also demonstrated potential for reducing populations of insect disease vectors, such as mosquitoes that transmit malaria to birds, people, and other species (McFarlane et al. 2018; Samuel et al. 2020).

#### 3.3.3. Integrative conservation approaches

The methods being developed and refined in de-extinction research, from ecological restoration via reintroduction of keystone species to genetic strategies for invasive species management, represent valuable additions to the conservation toolbox. By integrating lessons from successful rewilding initiatives with genome editing technologies and advanced reproductive techniques, conservationists can develop more holistic strategies that restore ecosystem functionality while safeguarding biodiversity. However, success requires careful consideration of modern ecological contexts and potential unintended consequences of genetic interventions.

### 3.4. Public engagement in conservation

In addition to the technological advances described above, de-extinction serves as a powerful catalyst for public engagement in conservation biology, in particular when it is presented as a complementary approach to more traditional conservation strategies. The prospect of resurrecting iconic species captures public imagination in ways that transcend traditional conservation messaging, creating new opportunities for funding, education, and international collaboration.

#### 3.4.1. Wonder and public imagination

De-extinction's ability to evoke awe and curiosity creates a "wonder factor" that serves as a gateway for broader public engagement (Rohwer and Marris, 2018; Sherkow and Greely 2013). Media coverage highlighting technological aspects may attract audiences not typically engaged in conservation issues. Some stakeholders may view reviving species lost to human activities as a form of ecological restoration. This narrative aligns with growing public interest in synthetic biology and genetic engineering, positioning de-extinction as a tangible application of cutting-edge science for environmental benefit. A 2023 Australian Broadcasting Corporation survey, for example, found that nearly 70% of respondents supported de-extinction and reintroduction of the thylacine, demonstrating strong public enthusiasm for reviving lost species (Zoe Kean and Lucie Cutting 2023).

In its de-extinction guidelines, the IUCN acknowledges that successful de-extinction and restoration ‘could provide a strong rationale for the preservation or restoration of habitat, and might be a means to reconnect people with the natural world in ways that could translate to conservation awareness beyond just the proxy’ (IUCN 2016). By making abstract concepts like biodiversity loss more concrete and actionable, de-extinction narratives humanize complex conservation challenges through charismatic species (Valdez et al. 2019).

#### 3.4.2. Funding dynamics and institutional support

The heightened visibility of de-extinction projects demonstrably affects conservation funding. High-profile initiatives attract philanthropic investments and corporate partnerships that might not otherwise support traditional programs (Jones 2014). This ‘halo effect’ extends beyond de-extinction itself, as funders develop increased interest in supporting broader ecosystem protection measures (Molhuizen et al. 2025, 13).

However, this landscape requires navigation to avoid resource imbalances or perceptions of de-extinction as a technological ‘quick fix’ for biodiversity loss (IUCN 2016; Valdez et al. 2019). Well-structured engagement initiatives can mitigate this risk by explicitly highlighting the high costs of biodiversity loss, thereby positioning de-extinction technologies within existing conservation frameworks rather than as competitors to them.

#### 3.4.3. International collaboration and policy innovation

De-extinction's transnational nature accelerates international cooperation in conservation governance. Reviving species from disparate biogeographic histories necessitates collaborative frameworks for genetic resource sharing, habitat restoration, and risk assessment. The IUCN's de-extinction guidelines exemplify how these challenges spur policy innovation, creating precedents for managing emerging biotechnologies in conservation contexts (IUCN 2016).

These collaborations often expose gaps in existing regulatory architectures. For instance, debates about de-extinction have prompted reevaluations of the Cartagena Protocol’s applicability to resurrected species, driving discussions about modernizing international biosafety agreements (Valdez et al. 2019). The technical demands foster unprecedented data sharing networks between geneticists, ecologists, and conservationists across borders.

#### 3.4.4. Educational opportunities

De-extinction's narrative power is particularly effective in STEM education, with opportunities to inspire new generations of conservation biologists. Integration into curricula provides multidisciplinary teaching opportunities combining genetics, ecology, and ethics. Museums and zoos capitalize on this interest through exhibits using de-extinction themes to explain broader concepts like trophic cascades and habitat connectivity.

This educational synergy emphasizes de-extinction’s capacity to function as both a scientific tool and a cultural touchstone, bridging the laboratory research and public engagement with biodiversity while making threats like climate change and habitat fragmentation more relatable to diverse audiences.

## 4. Outlook

De-extinction research has evolved from a speculative concept to a frontier of applied biotechnology, generating innovations that extend far beyond species revival. The genome engineering, advanced assisted reproductive technologies, and ecological modeling tools developed for de-extinction are providing new pathways for genetic rescue, species resilience, and ecosystem restoration. As these technologies mature, their integration into mainstream conservation will offer transformative approaches to biodiversity preservation.

Significant technical challenges remain. Fully reconstructing extinct genomes, developing reproductive strategies for functionally extinct species, and ensuring successful rewilding require continued innovation. Priority areas include refining genome editing precision and efficiency, optimizing cloning, and exogenous development protocols, and enhancing AI-driven ecological modeling for reintroduction planning. The field must also balance the compelling narrative of species resurrection with urgent protection needs for currently endangered species. The most immediate application of de-extinction technologies lies in using genetic interventions to prevent species from reaching extinction. This proactive approach avoids the complex challenges of ecosystem reintegration that extinct species face.

The future of de-extinction will be shaped by interdisciplinary collaboration across genomics, reproductive biology, conservation science, and ecological restoration. As genome editing becomes more precise and cost-effective, and as aART techniques advance, conservation applications will become increasingly feasible. AI and environmental monitoring advances will enable robust assessment of how reintroduced species interact with modern ecosystems, ensuring contributions to long-term ecological resilience rather than isolated scientific achievements. While it will be critical both to maintain a clear vision for how de-extinction technologies can serve conservation goals and to maintain a balance between technological ambition and ecological responsibility, de-extinction is increasingly understood as an emerging branch of conservation biology—one that complements and expands the existing toolkit.

## Supplementary Material

De-extinction_review_for_J_Heredity_v2-v1_tracked_changes_esaf069
